# The usefulness of a novel patient management decision aid to improve clinical decision-making skills in final year chiropractic students

**DOI:** 10.1186/s12998-019-0278-3

**Published:** 2019-09-19

**Authors:** Michael Hobbs, Dirk Crafford, Katherine MacRae, Anneliese Hulme, Stephney Whillier, Hazel Jenkins

**Affiliations:** Department of Chiropractic, Faculty of Science and Engineering, Macquarie University, Sydney, Australia

**Keywords:** Chiropractic, Management, Decision aid, Algorithm, Musculoskeletal, Conservative

## Abstract

**Background:**

The process of developing patient management plans requires a series of clinical decision-making skills that can take years in practice to develop. For the inexperienced practitioner, providing a logical, systematic patient management framework may assist in clinical scenarios and accelerate their decision-making skill development. The purpose of this study was to assess whether a novel clinical management decision aid would improve the management decision-making of chiropractic students.

**Methods:**

A prospective before and after study tracked chiropractic master degree students in their final year of study across a 10-week period from February–May, 2017. Case-based assessments were performed at baseline, after initial exposure to the decision aid, and after repeated exposure over the course of the semester. Outcome measures included the results from the 3 assessments, scored out of 20 by two markers using a standardised marking rubric, then averaged and converted to percentages; and 2 feedback questionnaires, given after initial exposure and at 10 weeks.

**Results:**

A total of 75 students (44 males; 31 females) participated in the study. The mean score at baseline was 8.34/20 (41.7%) (95% CI: 7.98, 8.70; SD: 1.56) and after initial exposure was 9.52/20 (47.6%) (95% CI: 9.06, 9.98; SD: 2.02). The mean score after repeated exposure was 15.04/20 (75.2%) (95% CI: 14.46, 15.62; SD: 2.54). From baseline to initial exposure, there was a statistically significant absolute increase in mean score of 1.18/20 (5.9%) (95% CI: 0.6, 1.76; *p* < 0.0001), or a 2.82/20 (14.1%) relative improvement. From baseline to repeated exposure, there was a statistically significant absolute increase in mean score of 6.7/20 (33.5%) (95% CI: 6.02, 7.38; *p* < 0.0001), or a 16.06/20 (80.3%) relative improvement. The questionnaire results were also favourable. 56/75 (75%) participants agreed that the decision aid was easy to use and 46/75 (61%) of participants agreed that the decision aid improved their ability to integrate various management techniques.

**Conclusion:**

Implementing a clinical management decision aid into the teaching curriculum helped to facilitate the ability of chiropractic students to develop patient management plans.

**Electronic supplementary material:**

The online version of this article (10.1186/s12998-019-0278-3) contains supplementary material, which is available to authorized users.

## Introduction

### Background

The process of developing individualised management plans in clinical practice requires a sequence of clinical decisions to integrate diagnostic and prognostic conclusions, best evidence, patient characteristics, and practitioner expertise [1, 2]. This is a skill that may take years to master. For inexperienced practitioners, offering a systemised approach to decision-making is useful to develop problem-solving skills until they are self-sufficient [3, 4].

Decision aids provide a sequential decision-making map, which can be used by clinicians to assist with problem-solving, such as selecting appropriate management strategies [1, 3–7]. Decision aids are commonly used in education to assist decision-making skill development [2–4, 7]. Clinicians using decision aids are provided with an explicit list of options, which helps clarify their thinking [2–4]. This is particularly useful for inexperienced practitioners, who tend to overestimate their level of competency and may be prone to overlooking important components of a patient’s management plan [7–10]. By providing a structure to assist in the integration of information from a variety of sources, a multifaceted patient-centred management plan can be developed.

Currently, there are no generic management decision aids for chiropractic clinical decision-making related to the application of appropriate intervention strategies [1, 5, 6, 11–17]. Whilst some decision aids do exist within the literature, they pertain to either diagnostic triaging or only address management for specific disorders. They do not attempt to encompass the broad spectrum of musculoskeletal disorders that would present to a chiropractic clinician [1, 5, 6, 11–17]. Also, to the best of our knowledge, no studies have attempted to assess the usefulness of the tool for chiropractors or chiropractic students [1, 5, 6, 11–17].

A management decision aid was designed via a consensus process by the chiropractic teaching staff at Macquarie University to facilitate student development of clinical management plans within the Master of Chiropractic degree (see Additional file 1). The aim was not to replace current guidelines and consensus documents, but rather to improve assimilation of information from a number of sources, and guide the decision-making processes.

The purpose of this study was to investigate whether the use of the developed management decision aid would influence students’ results in a series of three case-based assessments and whether the decision aid was seen as a useful and usable tool by students.

## Methods

### Study design and setting

A prospective before and after study was conducted on Master of Chiropractic students at Macquarie University, Australia from February–May 2017. Results were compared from 3 case-based assessments performed: at baseline, after initial exposure to the decision aid and after repeated exposures to the decision aid throughout the academic semester. Ethics approval was obtained from the Macquarie University Health and Research Ethics Committee, Reference Number 5201500894.

### Participants

#### Recruitment

All students enrolled in the final year of the Master of Chiropractic program at Macquarie University in 2017, who met inclusion criteria, were invited to participate.

The final year of the chiropractic Masters program is designed for students to simultaneously undertake the Chiropractic Diagnosis and Management course, involving tutorials and lectures focused on developing case management skills and the 12-month Clinical Internship program, in which students treat members of the public in one of the three university outpatient clinics. Potential participants were included if they were undertaking both the Chiropractic Management course and the Clinical Internship program for the first time. Participants had to be present for the first Chiropractic Diagnosis and Management tutorial, where the initial assessments were conducted.

Participation required written informed consent for the release of the students’ de-identified assessment marks and questionnaire feedback for group scoring analysis. Consent was run through during the first tutorial by the researchers, who were also co-students, but not necessarily in the class. Participants were given time to read through the information and it was made clear that no incentive or penalty was provided for participation or non-participation and that they were able to withdraw from the study at any time. The consent procedure was approved by Macquarie HREC.

The assessment tasks were built into the core curriculum to minimise stress and inconvenience to participants. Study participants were provided with a unique identifying number, which was recorded on the three assessments they performed. This allowed the tests to be de-identified for assessment purposes so assessors were blinded to student identity. It also allowed results between tests for each individual student to be prospectively followed and for re-identification of grades for formative feedback to the students and grading purposes in the unit. Tracking of results was performed by members of the research team not involved in the assessment process. Re-identification for release of final grades was performed by tutors who were involved in the teaching of the course, but were not members of the research team or involved in the assessment process.

It was determined that an absolute difference in assessment results of at least 5% would be necessary to demonstrate a meaningful change. This was determined by the research team based on that a 5% change would be a difference of 1 mark on a 20 point scale, and anything less than this was unlikely to be meaningful to the students. Power calculations were performed and a minimum of 40 participants were required to show a statistically significant difference with a 1/20 (5%) difference in assessment results.

### Data collection and clinical outcome variables

Participants completed written exams at three different time points to assess their ability to formulate suitable management plans when provided with history and examination findings of cases representative of typical chiropractic care. The first was a baseline assessment, performed prior to any exposure to the decision aid (see Additional file 2). Following completion of this assessment task, the decision aid was introduced and briefly explained to the participants. They were then asked to complete the second exam with the decision aid as a reference (see Additional file 3), so that the baseline and initial exposure assessments were both completed during the same two-hour tutorial time, with a short break in between. The third exam (see Additional file 4) was performed at ten weeks, after 9 weekly 2-h tutorials, during which students worked on clinical case management questions, using the decision aid to assist their learning. The decision aid was also referenced by the tutor throughout each tutorial.

A copy of the decision aid was not provided during this final exam. Participants were also asked to complete two anonymous questionnaires: one after the initial exposure to the decision aid (post-initial exposure questionnaire), and the other after repeated exposure (post-repeated exposure questionnaire) (see Additional files 5 and 6, respectively). Results of the two initial exams served as formative feedback with no effect on student grades. The final assessment result contributed towards the student’s formal grade for that unit.

The three assessments were independently marked by two assessors, who were part of the research team. The assessors were blinded to student identification and to which assessment tasks were baseline and initial exposure. One of the assessors was also blinded to the repeated exposure assessment. The other assessor, who was also involved in the teaching of the subject, could not be blinded to the repeated exposure assessment. The assessors followed a standardised marking rubric out of 20 (see Additional file 7). An average of the two assessors’ marks for each exam was calculated and converted to a percentage as the result for each participant. Assessment marks and questionnaire results were individually entered into Microsoft Excel by two authors, and checked for consistency. Discrepancies were corrected against the original.

The primary outcomes were the mean change scores between the baseline and the initial exposure assessments, and the baseline and the repeated exposure assessments. Secondary outcomes assessed the perceived usability and usefulness of the decision aid. After initial exposure to the decision aid, a questionnaire (see Additional file 5) was administered, using Likert style questions and free-text responses, and incorporating the System Usability Scale, originally designed by Brooke, 1996 [18], to assess the usability and ease of application of the decision aid (see Additional file 8). A second questionnaire was administered after the final assessment, using Likert style questions and free-text responses, to assess the perceived usefulness of the decision aid over the course of the 10 weeks of teaching (Additional file 6). The Likert style questions were scored 1–5, where 1 = strongly disagree, 2 = disagree, 3 = did not know how to respond, 4 = agree and 5 = strongly agree. These text anchors were provided verbally during the tutorials when the questionnaires were administered. Different questions were used for each questionnaire, as they were assessing different functions of the decision aid (the first questionnaire being primarily concerned with whether or not the decision aid was user-friendly and the second questionnaire being primarily concerned with whether or not the participants perceived the decision aid as useful for their learning).

### Data analysis

#### Objective results

Each participants’ score was calculated based on an average between the two assessors’ marks for their score out of 20 according to the marking rubric. Group mean scores were then calculated and converted to percentages with standard deviation and 95% confidence intervals. Group mean change scores were calculated between the baseline and initial exposure groups, and between the baseline and repeated exposure groups. A paired t-test, performed in Minitab® 18 (Minitab Pty Ltd., Sydney, Australia), was used to assess for a statistically significant difference between baseline and initial exposure, and baseline and final assessment.

#### Subjective results

Likert scale responses in the questionnaires were analysed descriptively. These were grouped into those that agreed (4 or 5 on the Likert scale), didn’t know how to respond (3 on the Likert scale), or disagreed (1 or 2 on the Likert scale) with the statement. Qualitative analysis of written feedback from the final questionnaire was performed independently by two authors, with responses grouped into common themes.

Analysis of the usability of the decision aid was completed using the System Usability Scoring (SUS) scale formula, applied to the first 10 questions of the post-initial exposure questionnaire [18, 19]. The SUS scale measures the subjective usability of a particular tool, taking into consideration its effectiveness, efficiency and satisfaction [18–21]. The SUS score produced was an average of the individual scores. It was interpreted using an adjective rating scale developed by Bangor et al., 2008 [19].

## Results

### Participants

Of 85 students enrolled in Chiropractic Management in 2017, 75 met inclusion criteria, all of whom consented to being included in the study. Ten students did not meet inclusion criteria: three were not formally enrolled in both subjects, four were absent during week 1 and three were primary researchers, whose results were withheld due to potential bias. No students withdrew from the study at any stage. Of the participants, 44 were male (58.7%), and the mean age was 24 years (range 20–45 years).

### Results of assessments

**Fig. 1:**
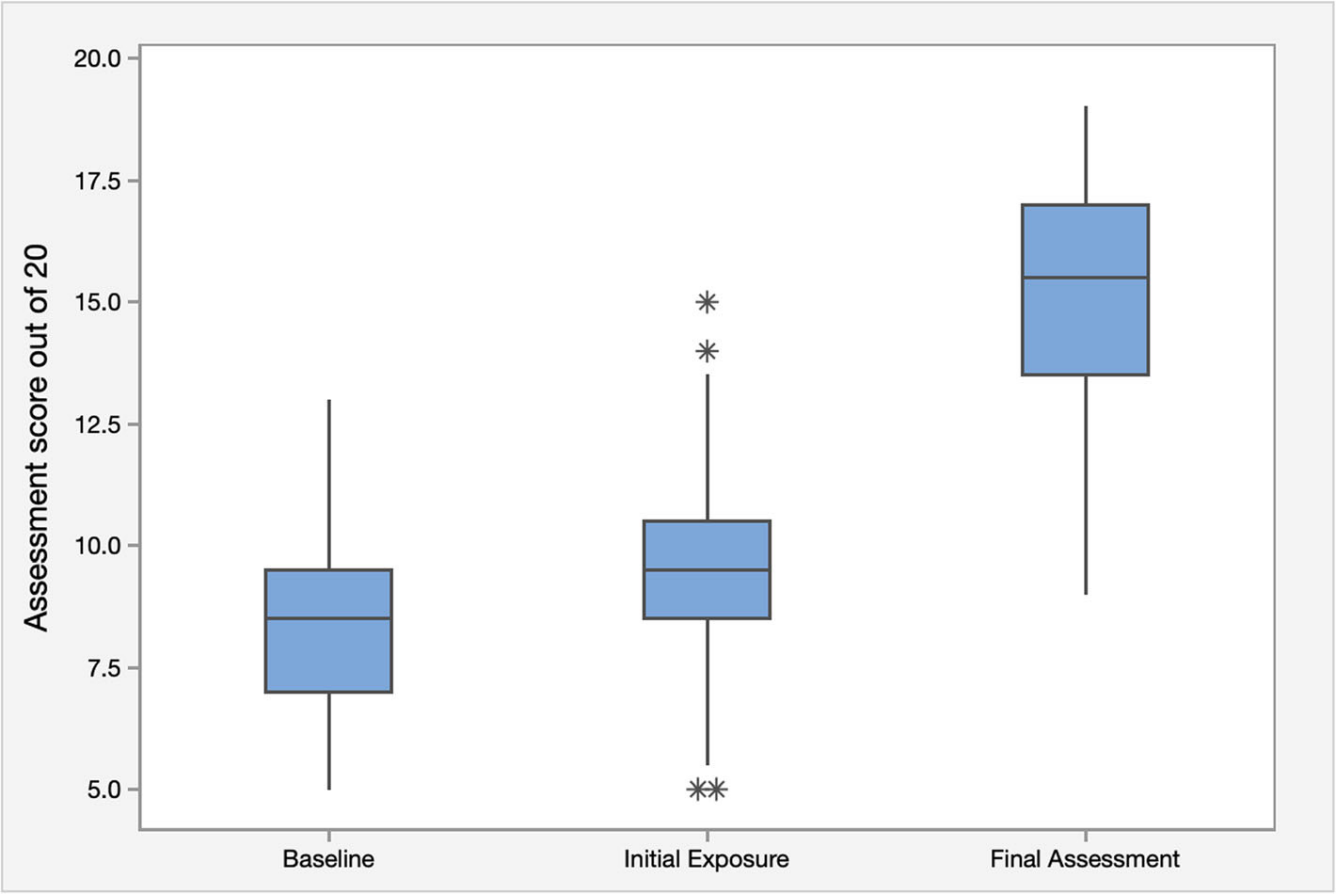
Mean results and score ranges of assessment score percentages performed at baseline, after initial exposure and after repeated exposure

**Fig. 2:**
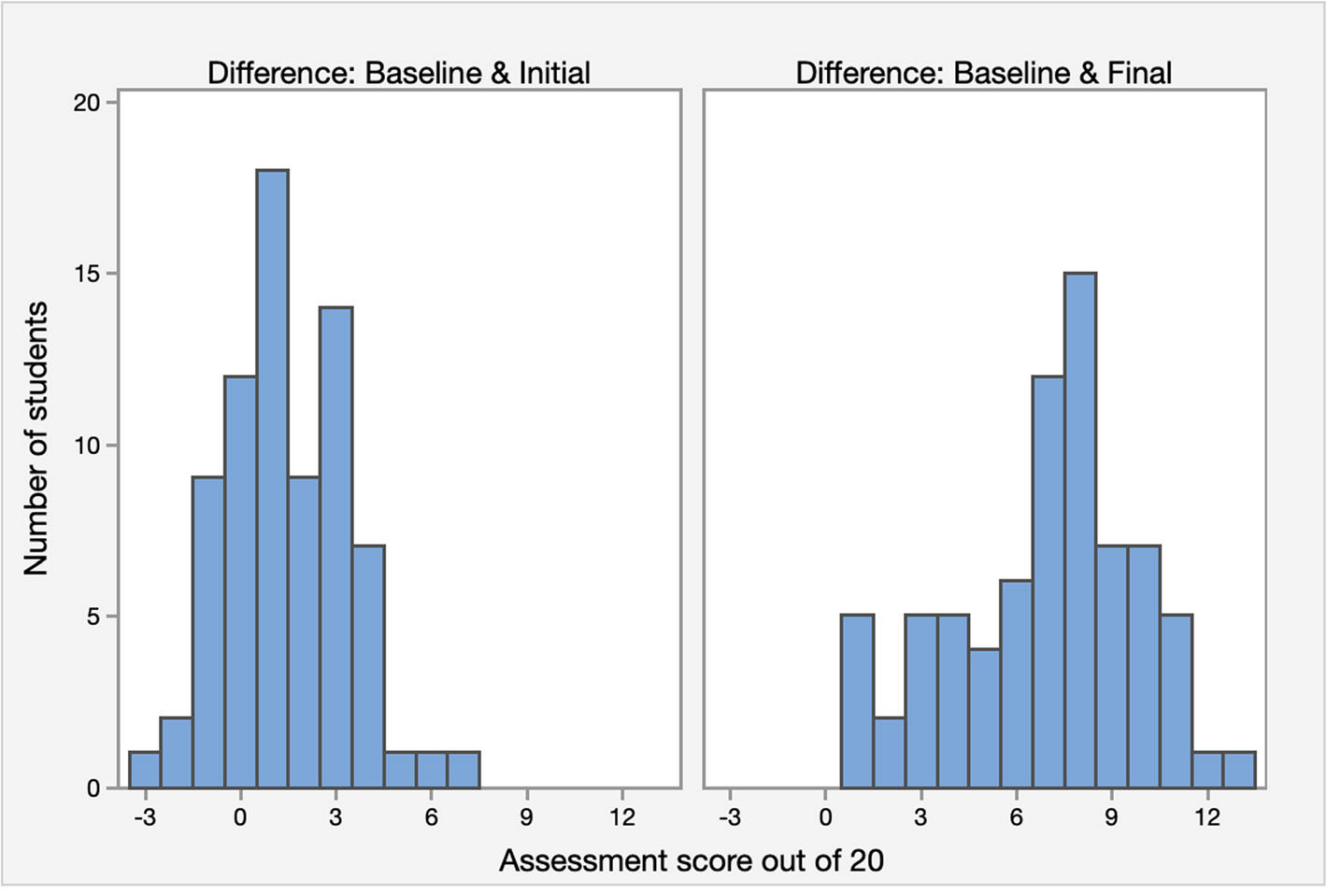
Histogram of absolute difference in assessment score percentages between baseline and initial exposure scores and baseline and repeated exposure scores

The results of the three case-based assessments using the standardised scoring rubric are presented in Figs. 1 and 2. The mean score at baseline was 8.34/20 (41.7%) (95% CI: 7.98, 8.70; SD: 1.56), with a range of scores from 5 to 13/20 (20–65%). The mean score after initial exposure was 9.52/20 (47.6%) (95% CI: 9.06, 9.98; SD: 2.02), with a range of scores from 5 to 15/20 (20–75%). The mean score after repeated exposure was 15.04/20 (75.2%) (95% CI: 14.46, 15.62; SD: 2.54), with a range of scores from 9 to 19/20 (45–95%).

#### Changes in assessment results between groups

From baseline to initial exposure, there was a statistically significant absolute increase in mean score of 1.18/20 (5.9%) (95% CI: 0.6, 1.76; *p* < 0.0001), or a 2.82/20 (14.1%) relative improvement. From baseline to repeated exposure, there was a statistically significant absolute increase in mean score of 6.7/20 (33.5%) (95% CI: 6.02, 7.38; p < 0.0001), or an 16.06/20 (80.3%) relative improvement. The range of changed scores between groups are presented in Fig. 2. From baseline to initial exposure, 12 participants (16%) had a decrease in results, 12 participants (16%) maintained the same mark, and 51 participants (68%) had an increase in results. The spread ranged from an absolute decrease of 3/20 (15%) to an absolute increase of 7/20 (35%). From baseline to repeated exposure, all participants had an absolute increase in results, ranging from 1 to 13/20 (5–65%).

### Post-initial exposure questionnaire results

#### Quantitative data

**Fig. 3:**
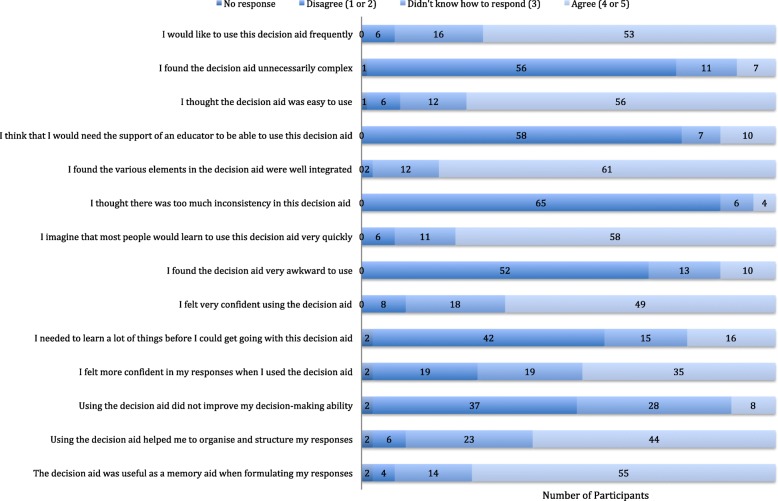
Descriptive analysis of post-initial exposure questionnaire results

A descriptive analysis of the results are presented in Fig. 3. 56/75 (75%) of participants agreed that the decision aid was easy to use. 49/75 (65%) agreed that they felt very confident using the decision aid. The majority of participants (52/75 or 69.3%) disagreed (ie. scored 1 or 2) that the decision aid was awkward to use, needed the support of an educator (58/75 or 77.3%), or was unnecessarily complex (56/75 or 74.7%). 55/75 participants (73%) agreed that the decision aid was useful as a memory aid and 35/75 participants (47%) felt more confident in their responses when they used the decision aid.

#### Qualitative feedback

29/75 participants (38.7%) provided written feedback. 24/75 participants (32%) responded that they found it a useful tool. 4/75 participants (5.3%) responded that they struggled to implement the tool and 2/75 participants (2.7%) stated that it wasn’t useful as it was provided too late in the degree.

#### System usability score

The mean System Usability Score was 73.7, which is considered within the ‘acceptable’ range [19, 21]. The median SUS score was 77.5 and the interquartile range 21.25 (Q1 = 63.75; Q3 = 85).

### Post-repeated exposure questionnaire results

#### Quantitative data

**Fig. 4:**
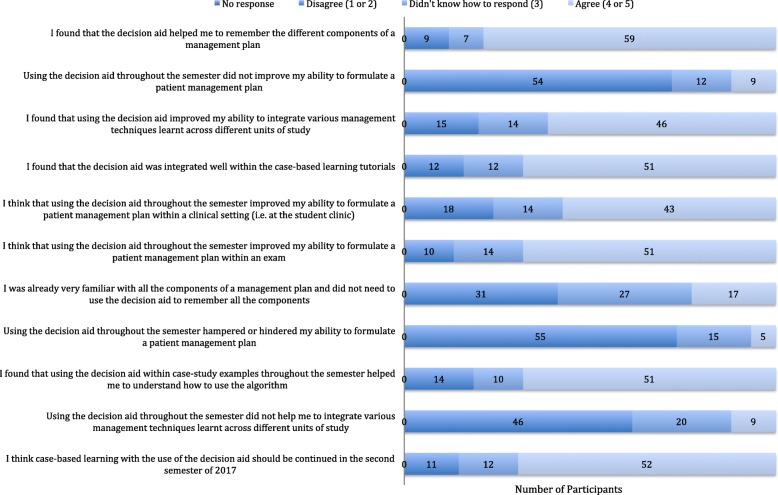
Descriptive analysis of post-repeated exposure questionnaire results

Descriptive analysis of the results of the final questionnaire are presented in Fig. 4. 59/75 participants (79%) agreed that the decision aid helped them to remember the different components of a management plan. 46/75 participants (61%) agreed that the decision aid improved their ability to integrate various management techniques. 51/75 participants (65%) agreed that the decision aid improved their ability to formulate management plans within an exam; whilst 43/75 (57%) agreed that it improved their ability to formulate management plans within the student clinic. 55/75 participants (73%) disagreed that using the decision aid hampered or hindered their ability to formulate a patient management plan and 52/75 (69%) agreed that case-based learning with the use of the decision aid should be continued in the second teaching semester.

#### Qualitative feedback

The majority of participants responded that they had found the decision aid helpful in developing their clinical decision making skills. 52/75 participants (69%) commented that the most helpful aspect was providing a structure for management decision-making. 11/75 participants (15%) commented that it aided as a memory tool. Ten responses (13%) stated the decision aid had not been helpful; the main reason cited by half of these when asked what could be improved or what was missing was that it did not translate well into clinical settings as it lacked the complexity to accommodate for individual case characteristics. When asked what was missing or what could be improved, 12/75 respondents (16%) commented that specific examples would have been useful. 5/75 participants (7%) also commented that it could be improved if it considered the progression of patient management plans over time. Interestingly, 14/75 (19%) participants commented that the decision aid needed to be integrated earlier into the curriculum.

## Discussion

This research examined the usefulness of a novel decision aid for chiropractic students in assisting their development of clinical decision-making skills.

A statistically significant relative improvement of scores occurred both after initial exposure (2.82/20 (14.1%), *p* < 0,0001) and repeated exposure (16.06/20 (80.3%), *p* < 0.0001). The absolute improvements of 1.2/20 (6.0%) and 7.1/20 (35.5%) respectively were both greater than the 1/20 (5%) absolute change initially considered as a meaningful change in results. The decision aid was perceived to be both usable (mean SUS score 73.7) and useful, the majority of participants reporting that it served as a memory-aid whilst helping to integrate different management techniques to develop a management plan. These findings suggest that the clinical management decision aid may have been useful in facilitating an improvement in student ability to formulate a patient management plan on immediate exposure, and that this improvement was increased on repeated exposure, which may have been at least in part due to repeated exposure to the decision aid.

### Strengths and limitations

The decision aid was assessed at three time points to allow determination of: 1) A baseline score, prior to the students using the decision aid or being involved in clinical teaching; 2) A score after initial exposure to the decision aid only, and without any involvement in clinical teaching; and 3) A score after repeated use of the decision aid in a clinical context. A statistically significant increase in mean scores was observed after initial exposure to the decision aid only, suggesting that use of the decision aid even without any further clinical teaching may have contributed to an improvement in students’ ability to formulate a patient management plan. The perceived usefulness and usability of the tool was also considered important to measure in addition to improvement in student marks, as these factors would impact student use of the decision aid. These concepts were tested both at initial exposure and after repeated exposure, and students generally reported the decision aid to be both useful and usable.

Testing the usefulness of decision aids is a relatively emerging field of research when it comes to healthcare education [22, 23]. Determining what is considered an appropriate rating of usability and usefulness remains uncertain, with significant heterogeneity in how studies measure usefulness [22, 23]. Furthermore, what constitutes an appropriate rating for effecting curriculum implementation is still debatable. Nevertheless, decision aids have been shown to be considered useful by students for developing their decision-making skills [22, 23]. This study may be useful in contributing to these important conversations around educational efficiency and the tools we implement to assist in the development of clinical decision-making.

Limitations of this study include the study design, variability in assessment cases, and the inability to completely blind all assessors. A randomised controlled trial would have constituted the ideal study design to test the efficacy of the implementation of a management decision aid. However, this was not possible due to the involvement of the participants in a teaching program and the potential academic disadvantage to the control group, either real or perceived. The before and after design used was the best alternative, that would allow for baseline control measurements, but not represent any perceived academic disadvantage to the students.

Due to teaching limitations, the baseline and initial exposure assessments were performed in the same two-hour tutorial time with a short break in between the assessments. This may have led to exam fatigue potentially resulting in poorer results in the second assessment and an underestimation of the effects of initial exposure to the decision aid.

Cases assessed at each time point were necessarily different to prevent an improvement in marks due to learning from repeated exposure to the same case. Therefore, it is possible that some change in marks was related to the different case being presented and not to the use of the decision aid. All of the selected cases were standardised as much as possible and were chosen as musculoskeletal presentations that would commonly present in chiropractic care and which students had received prior exposure to. The variability in cases or the potential for exam fatigue may explain the 12 participants (16.0%) who performed worse after initial exposure to the decision aid than they did at baseline.

An additional limitation of the study design was the inability to determine whether improvements in the final repeated exposure assessment were solely related to use of the decision aid. Increased clinical experience, familiarity with marking criteria, formative feedback from the previous assessments, and increased incentive to perform well in the final assessment due to inclusion of results in the unit grade may all have contributed to the improvement seen. The final questionnaire, however, indicated that the majority of participants had found the decision aid to be useful in management plan development both within the exam and in clinical situations even with all the other factors outlined above.

Complete blinding of the time point of the assessments being marked could not be achieved for one of the two assessors. The baseline and initial exposure assessments were marked immediately after the assessment so that formative feedback could be provided to the students in line with teaching requirements for the unit. The assessor marking these was blinded to which case was baseline and which was initial exposure, but could not be blinded to the repeated exposure assessment conducted later in the semester. A structured marking rubric was used to limit bias as much as possible. To further limit bias, an independent marker assessed all three assessments after completion of the repeated exposure assessment and was completely blinded to the assessment timing. Results from the two assessors were averaged before statistical analysis was performed, and analysis of the results from each assessor found similar trends in results and statistically significant mean differences.

The SUS is a useful usability assessment tool as it is easy to administer, calculate and interpret [19, 21]. It is considered a reliable indicator of usability, however what constitutes a ‘good’ SUS score remains debatable [19, 21]. Bangor et al., 2008, reviewing 2324 surveys over 206 studies, determined that the mean SUS study score across studies was 69.69 (SD: 11.87) and thus suggested a score above 70 as being within the ‘acceptable’ range [19]. However, the ‘acceptable’ range may still vary for different disciplines. Due to the lack of research, it is difficult to know what would be considered an industry-specific ‘acceptable’ range. Future analysis of teaching aids using the SUS may be beneficial in order to arrive at a consensus as to what is deemed ‘acceptable’ by educational standards both for student use and for driving curriculum change.

The SUS could also be confusing and frustrating to some, due to the scoring system in relation to negative questions. There is a possibility that some participants forgot to reverse their answers. Nevertheless, it is common in psychometric assessments to vary the tone of the questions in order to reduce acquiescent bias. The SUS could have been improved by including the 7-point adjective rating scale as proposed by Bangor et al., 2008, which has shown to very closely match the SUS score [19]. An opportunity for descriptive feedback was instead offered in both questionnaires, which provided more detail as to the reasoning behind the participants’ responses. This was important to include, as the SUS in itself does not provide feedback about what was needed to improve.

The limitations of testing for perceived usability must also be acknowledged. Participants are only reporting on their subjective experience of the interaction with the aid, which may be biased and does not necessarily correlate with an objective improvement in assessment scores. It may have been appropriate to analyse the relationship between objective scores and participants’ subjective feedback of using the decision aid, however this was not done due to feedback remaining anonymous.

### Implications, outcomes and significance

The use of the decision aid within the teaching of clinical decision-making may facilitate student development of appropriate management plans. Clinical decision-making skills typically take time to develop, and this decision aid may be useful to provide a structure to the students’ decision-making process until further clinical experience can be obtained. The use of a decision-making aid may accelerate the inexperienced clinician’s ability to provide complete management plans and minimise the likelihood of overlooking important aspects of care. This decision aid may also be useful to help standardise care, improve patient safety and enhance patient outcomes by facilitating co-management where appropriate. A follow-up RCT would be most appropriate to assess the potential implications of this decision aid.

With further research, integration of the decision aid may be recommended into the teaching of chiropractic clinical management. Earlier integration of a decision aid that incorporates diagnosis and management into chiropractic programs may also be useful to allow integration of new information through a consistent structure throughout the academic years.

Although use of the decision aid was associated with improved performance, limitations have been recognised and further development of the decision aid may be indicated. In particular, 15/75 (20%) of participants disagreed with the statement that the decision aid helped to integrate different management techniques, and 18/75 (24%) disagreed with the statement that the decision aid helped to develop management plans in a more clinical context. Usability, whilst acceptable, could also still be improved. Further attempts to develop the decision aid to improve the ability to deal with individualised patient cases may improve the use of the decision aid in a clinical context. It may also be that these students were further developed in their clinical decision-making, and so the decision aid, designed for students of lesser decision-making ability, was not as relevant. Due to the study design and anonymity of the questionnaires, correlation testing between perceived acceptability of the decision aid and performance could not be performed.

The decision aid was only tested as it pertained to model clinical scenarios, however testing it in clinical settings with real patients would also be recommended. Although 51/75 (68%) of participants found the decision aid improved their ability to formulate management plans within an exam, only 43/75 (57%) of participants found the decision aid improved their ability to formulate management plans within a clinical setting. 18/75 (24%) of participants disagreed that the decision aid improved their ability to formulate a management plan within a clinical setting. Five responses from the post-repeated exposure questionnaire also commented that they found it useful for exam settings only and not with real patient management. Thus, the usefulness of the decision aid for guiding decision-making about living patients should not be concluded based on these study results alone. Future studies may want to also assess patient experience of the decision aid, not only student experience.

## Conclusion

The use of the management decision aid was associated with an improved ability to develop patient management plans in an examination setting, and was seen as a useful and usable tool by students both after initial and repeated exposures. The use of a decision aid within chiropractic clinical teaching offers a structural framework to aid inexperienced clinicians in the development of clinical decision-making. It may further facilitate standardised care for chiropractic patients by ensuring these clinicians are better equipped for evidence-based practice.

## Additional files

Additional file 1: The management decision aid. (PDF 395 kb)

Additional file 2: Case-based assessment task at baseline. (PDF 84 kb)

Additional file 3: Case-based assessment task after initial exposure to decision aid. (PDF 83 kb)

Additional file 4: Case-based assessment tasks after repeated exposure to decision aid (three cases used). (PDF 113 kb)

Additional file 5: Post-initial exposure questionnaire. (PDF 72 kb)

Additional file 6: Post-repeated exposure questionnaire. (PDF 84 kb)

Additional file 7: Standardised marking rubric for case-based assessment tasks. (PDF 122 kb)

Additional file 8: Interpreting the System Usability Scale (SUS) score according to Bangor et al., 2008 (reproduced with permission) [19]. (PDF 77 kb)

## Data Availability

All study materials used during this study are included in this published article. Access to the full datasets including individual results from the 3 case-based assessments and 2 questionnaires completed during this study is available from the corresponding author on reasonable request.
