# Supplementary material for: The usefulness of a novel patient management decision aid to improve clinical decision-making skills in final year chiropractic students
Source: Chiropr Man Therap. 2019 Sep 19;27:55. doi: 10.1186/s12998-019-0278-3 (PMC6751823; doi:10.1186/s12998-019-0278-3)
Supplement: Supplementary file 1 — The management decision aid. (PDF 395 kb) [file 12998_2019_278_MOESM1_ESM.pdf]

Additional File 1

The Management Decision Aid

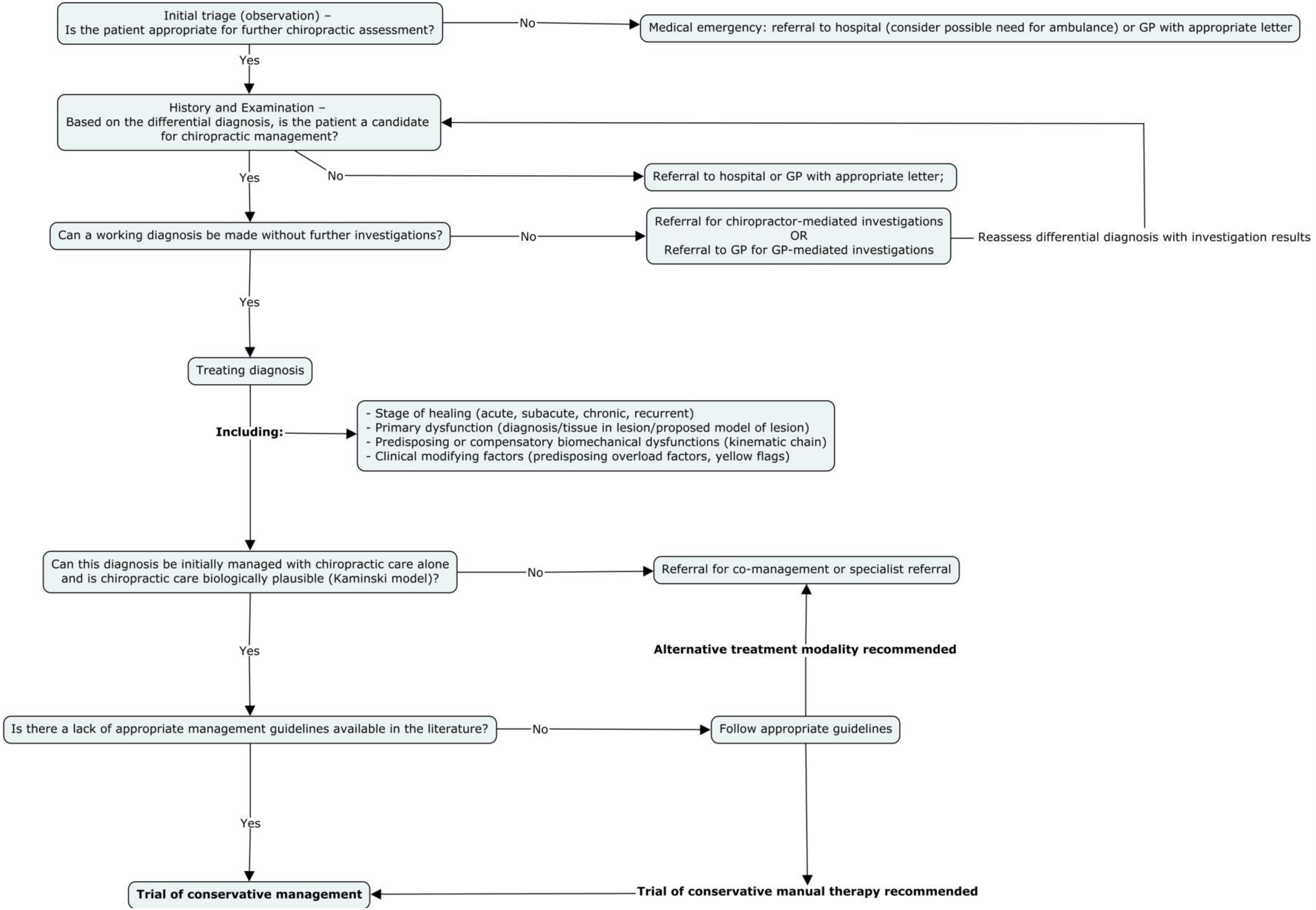

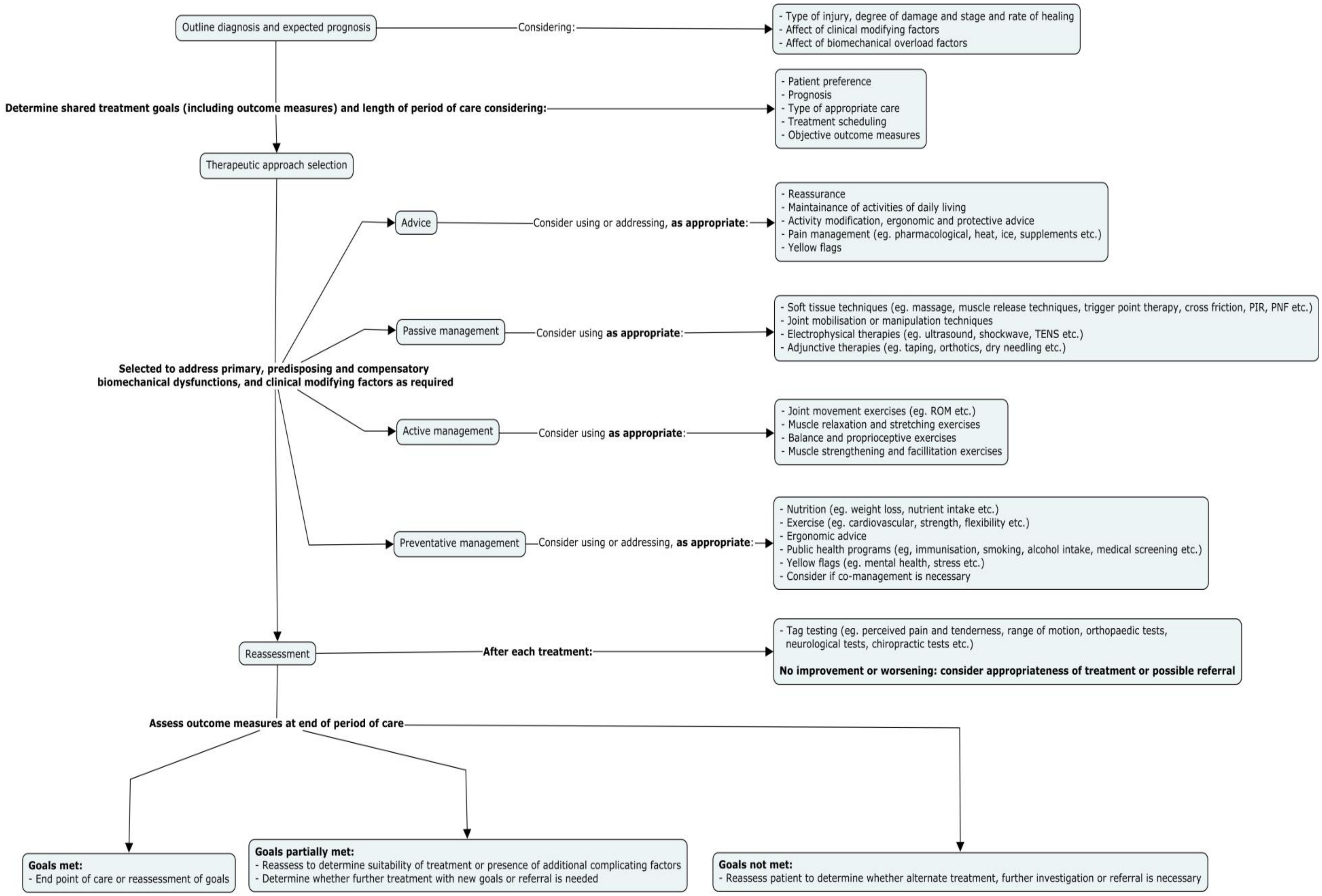
Outline diagnosis and expected prognosis
Considering:
- Type of injury, degree of damage and stage and rate of healing
- Affect of clinical modifying factors
- Affect of biomechanical overload factors
Determine shared treatment goals (including outcome measures) and length of period of care considering:
- Patient preference
- Prognosis
- Type of appropriate care
- Treatment scheduling
- Objective outcome measures
Therapeutic approach selection
Selected to address primary, predisposing and compensatory biomechanical dysfunctions, and clinical modifying factors as required
Advice
Consider using or addressing, **as appropriate**:
- Reassurance
- Maintainance of activities of daily living
- Activity modification, ergonomic and protective advice
- Pain management (eg. pharmacological, heat, ice, supplements etc.)
- Yellow flags
Passive management
Consider using **as appropriate**:
- Soft tissue techniques (eg. massage, muscle release techniques, trigger point therapy, cross friction, PIR, PNF etc.)
- Joint mobilisation or manipulation techniques
- Electrophysical therapies (eg. ultrasound, shockwave, TENS etc.)
- Adjunctive therapies (eg. taping, orthotics, dry needling etc.)
Active management
Consider using **as appropriate**:
- Joint movement exercises (eg. ROM etc.)
- Muscle relaxation and stretching exercises
- Balance and proprioceptive exercises
- Muscle strengthening and facillitation exercises
Preventative management
Consider using or addressing, **as appropriate**:
- Nutrition (eg. weight loss, nutrient intake etc.)
- Exercise (eg. cardiovascular, strength, flexibility etc.)
- Ergonomic advice
- Public health programs (eg, immunisation, smoking, alcohol intake, medical screening etc.)
- Yellow flags (eg. mental health, stress etc.)
- Consider if co-management is necessary
Reassessment
After each treatment:
- Tag testing (eg. perceived pain and tenderness, range of motion, orthopaedic tests, neurological tests, chiropractic tests etc.)
No improvement or worsening: consider appropriateness of treatment or possible referral
Assess outcome measures at end of period of care
Goals met:
- End point of care or reassessment of goals
Goals partially met:
- Reassess to determine suitability of treatment or presence of additional complicating factors
- Determine whether further treatment with new goals or referral is needed
Goals not met:
- Reassess patient to determine whether alternate treatment, further investigation or referral is necessary
