# Supplementary material for: The usefulness of a novel patient management decision aid to improve clinical decision-making skills in final year chiropractic students
Source: Chiropr Man Therap. 2019 Sep 19;27:55. doi: 10.1186/s12998-019-0278-3 (PMC6751823; doi:10.1186/s12998-019-0278-3)
Supplement: Supplementary file 2 — Case-based assessment task at baseline. (PDF 84 kb) [file 12998_2019_278_MOESM2_ESM.pdf]

Additional File 2

Case-Based Assessment Task at Baseline

Case Study

Peter is a 45 year old male who presents with neck pain and headaches. He reports that he has experienced recurrent low intensity neck pain/discomfort for many years. He first experienced the headache 4 weeks ago. Past episodes of neck pain have usually resolved by themselves over a few days and having a hot bath or shower has usually helped to resolve the pain. Over the past 4 weeks, the neck pain episodes have lasted longer than previously. Although hot water still helps the symptoms, the pain hasn't gone completely. The neck pain is described as a dull ache bilaterally in the upper neck with some aching pain spreading down the right side of the neck towards the top of the shoulder. The ache also spreads up bilaterally around the skull towards the temples to give the headache he has been experiencing over the last 4 weeks. The neck pain and headache seem to be linked with each other and both get worse at the end of the day. He reports that both the neck pain and headaches are less intense in the morning, but he can still feel some residual symptoms and knows that 'it is not quite right'.

Peter is a carpenter by trade with his own business and often spends many hours bending over the project he is working on. In the last 4 weeks he has found that particularly bending his head forward for longer periods of time increases his symptoms to the point where he finds it difficult to keep working in that position. Changing position does help relieve the pain but nothing alleviates it entirely once it has started. He has also noted that doing a lot of work with his right arm has started to increase his neck pain as well. Peter reports that when his neck pain and headaches are at their worst he would rate them as 6/10 on a numerical pain scale (NPS) and in the morning they decrease to a 1/10 on a NPS. Although he says that lying down and going to bed does help to decrease the pain he has not been sleeping very well lately. He thinks this is related to increased stress and physical work in his business, where one of his full-time employees recently left, but he hasn't been able to find a replacement to cover the workload.

Peter reports that he is generally physically active but hasn't been exercising as much over the last month due to the increase time spent at work. He is married with 2 children aged 8 and 10 and reports

no issues in his home life. He hasn't been to the doctor for many years and can't think of any issues in his past medical history.

Examination

General observation	Peter is a fit looking male. He doesn't appear to be in any particular discomfort with general movement
Vitals	Blood pressure: 130/85 Heart rate: 78 bpm
Postural analysis	Flattening of lumbar lordosis and thoracic kyphosis<br>Increased upper thoracic kyphosis from T3 and forward head carriage with hyperextension in the upper cervical spine Right shoulder hiking with gothic shoulder appearance
Range of motion	Cervical spine: decreased flexion to 40 degrees with increase in neck ache and headache; decreased left lateral flexion to 20 degrees with pulling through the right shoulder<br><br>Thoracic spine: normal<br>Lumbar spine: decreased lumbar extension to 5 degrees, no pain<br>Left and right shoulders: normal
Motion palpation	Occiput restricted into flexion<br>C1 restricted in right lateral flexion and left rotation<br>C7 restricted into flexion<br>T3 restricted in extension<br>T11 restricted in extension<br>L4 restricted in P-A glide and right rotation

Static palpation	Bilateral suboccipital muscles hypertonic and tender with pain referral into the head on palpation<br>Upper trapezius hypertonic bilaterally but tender on the right<br>Anterior scalenes hypertonic and tender, worse on the right

Orthopaedic tests	Cervical compression negative<br>Cervical distraction produced some mild upper neck discomfort<br>Cervical kemps negative
Neurological tests	Cranial nerve exam normal except for mild weakness of the right upper trapezius (muscle strength 4/5) compared to the left (muscle strength 5/5) with increased discomfort in the neck
Functional tests	Chin jut at 2 seconds on Julls test and discomfort noted in the neck during the test<br>Elevation of the scapula at 20 degrees of arm abduction in the arm abduction movement pattern on the right; elevation of the scapula at 50 degrees of arm abduction on the left<br><br>Wall angel movement produced neck pain when performing the chin tuck, correct positioning maintained<br>Trunk flexion movement pattern demonstrated chin jut and inability to maintain the heels on the couch until the scapulae had cleared the bench

Peter was given the following diagnosis:

Subacute tension headaches with associated suboccipital and right upper trapezius muscle spasm. Predisposed by forward head carriage and upper thoracic extension restriction, and underactive deep neck flexor and lower trapezius muscles. Complicated by work posture, stress and a decrease in normal activity.

Question 1:

Based on the above information, please outline a detailed 6-week management plan for this patient.
