# Supplementary material for: The usefulness of a novel patient management decision aid to improve clinical decision-making skills in final year chiropractic students
Source: Chiropr Man Therap. 2019 Sep 19;27:55. doi: 10.1186/s12998-019-0278-3 (PMC6751823; doi:10.1186/s12998-019-0278-3)
Supplement: Supplementary file 3 — Case-based assessment task after initial exposure to decision aid. (PDF 83 kb) [file 12998_2019_278_MOESM3_ESM.pdf]

Additional File 3

Case-based Assessment Task After Initial Exposure to Decision Aid

Case Study

Richard, a 42-year-old accountant presents with a 4-week history of low back pain. He reports that initially he felt his back 'go' when he got out of bed in the morning. The pain at that time was quite severe and he couldn't straighten up properly or move easily. He took the day off work and rested. The pain was slightly easier the next day and he felt like he could go back to work. Although the pain has decreased over the last 4 weeks he still feels a general ache in the low back and that he can't move as easily as normal. He is now concerned that the pain has not completely resolved, as he has had similar previous episodes where the pain has completely resolved within a few days.

The initial pain was focused on the right side of his low back with some referral into the right upper buttock. Now the pain is more central at the bottom of his low back with referred pain still more predominant into the right upper buttock. He describes the pain as an ache, measuring 6/10 on a VAS at worst. He reports the pain as worst when he needs to stand still for longer than 30 minutes, which he regularly does at his sons' soccer training and games. Although sitting does not seem to aggravate the pain he doesn't feel comfortable sitting for longer than an hour and he finds it more difficult to get up from his chair than normal. He feels that he needs to use his arms to push himself out of the chair because he feels his back is weaker than normal and he can't move as easily. Once he gets moving he generally feels better but when the pain is bad he finds that only sitting or lying down help to relieve it. He took some nurofen on the first day of pain which gave some mild relief but he hasn't taken anything since then.

Richard normally exercises three times per week with a combination of running and weights training. He hasn't exercised since the onset of pain as he is worried he will make the back pain worse. He can't think of anything that might have initiated the back pain and he has only had 2 prior occurrences of pain, both over 5 years ago. Richard doesn't report any other symptoms with no pain, pins and needles, numbness or weakness of either lower extremity. He reports his sleep has not been disturbed except for the first night where it was hard to find a comfortable position. However, he reports that his back is a bit achy when he wakes in the morning before he gets moving. He generally sleeps flat on his back.

Richards systems review and medical history were unremarkable. He is married with 2 children: a 9 year old boy and a 7 year old girl. He is generally happy in both his job and home life.

Richards examination results are outlined in the table below:

Examination:

Postural analysis	Anterior drawn posture with an increased lumbar lordosis; Right elevated pelvis; Decreased thoracic kyphosis; Low right shoulder; Forward head carriage; Bilateral foot pronation; Reduced muscle tone bilateral gluteal muscles; Bulking of the thoracolumbar erector spinae muscles bilaterally (worse on the right); Increased angulation of the left upper trapezius muscle
Range of Motion	Lumbar: Decreased flexion with pulling through the low back and hamstrings; Pain on end-range extension in the central low back; Decreased left lumbar rotation and lateral flexion but no pain Hip: Reduced right hip flexion and flexion with adduction but no pain
Muscle assessment	Shortness of the bilateral gastrocnemius, bilateral hamstrings, bilateral psoas, bilateral lumbar erector spinae, right quadratus lumborum and left upper trapezius muscles Tenderness to palpation and hypertonicity of the bilateral lower erector spinae muscles and right gluteus medius muscle
Joint assessment	Tenderness on prone springing of L5 and T12 vertebrae<br>Joint restrictions: L5 left rotation and left lateral flexion restriction; T12 extension restriction; T4 flexion restriction; C2 right rotation and left lateral flexion restriction; Right hip lateral glide restriction

Orthopaedic/Neurologic assessment	Compression/distraction: negative Lumbar kemps: positive for pain on right<br>Slump: negative<br><br>SLR: limited to 70 degrees bilaterally but no pain or symptoms<br>Gaenslens: negative<br>Fabere: negative<br><br>Hip scour test: right hip restricted but no pain
Functional assessment	Hip extension movement pattern: slow activation of gluteals bilaterally with overactivity of hamstrings and lumbar erector spinae (worse on right)<br>Active straight leg raise: pelvic torsion noted bilaterally (worse on right)<br>Hip abduction: early hike of pelvis on right; left normal

Richard was given the following diagnosis:

Subacute right L5 facet syndrome with associated lumbar erector spinae and right gluteus medius muscle spasm. Predisposed by thoracolumbar extension restriction and imbalance between the overactive erector spinae and weak gluteal and abdominal muscles. Complicated by a decrease in normal activity and sedentary profession.

Question 1:

Based on the above information, outline a detailed 6-week management plan for this patient
