# Supplementary material for: The usefulness of a novel patient management decision aid to improve clinical decision-making skills in final year chiropractic students
Source: Chiropr Man Therap. 2019 Sep 19;27:55. doi: 10.1186/s12998-019-0278-3 (PMC6751823; doi:10.1186/s12998-019-0278-3)
Supplement: Supplementary file 4 — Case-based assessment tasks after repeated exposure to decision aid (three cases used). (PDF 113 kb) [file 12998_2019_278_MOESM4_ESM.pdf]

Additional File 4

Case-based assessment tasks after repeated exposure to decision aid (3 cases used)

Case Study 1

Paul, a 42-year-old IT consultant presents with a two week history of left hip and groin pain. Paul first felt pain in his groin during a weeknight touch football game two weeks ago. He felt the muscles in his inner groin catch on the left side as he was stepping around another player. At first he thought he had injured himself but he found that he was able to keep playing the rest of the game. A little while after the game, however, he found that the area stiffened up with an ache in the medial hip/groin region, and that the pain would become sharp when he went to move in different directions.

Since then the pain has decreased slightly but he still gets quite stiff and achy in the left medial hip, especially first thing in the morning or when he has been sitting for extended periods. The sharper pain still comes on with certain movements, particularly when he first starts walking, or walking up or down stairs. He hasn't tried running since the injury and hasn't played any touch football since then. He reports that he has a state competition for touch football coming up in a couple of months and he wants to make sure that he can play in that. Paul rates the achy pain as a 4 out of 10, increasing to a 6 out of 10 with the sharp exacerbations. There is no pain referral and no associated numbness, pins and needles, or weakness. Paul reports that once he gets his hip in the right position he is comfortable in bed but it is painful if he turns onto his right hand side. Although he's not in too much discomfort he has been having difficulty sleeping, but reports this has been ongoing over the last couple of months due to increased stress at work.

Paul used to be very active, playing touch football or rugby several times a week, and running regularly as well. Over the last few years, however, he has not been able to find as much time for exercise and has only been able to play touch football once a week. He stopped rugby when he turned 40 as felt he was getting too old to keep playing. He feels that he has lost condition and has started to put on some weight. He has had previous injuries due to sport but usually around his ankles or knees. None of the previous injuries were serious and they generally recovered well with minimal treatment. He is concerned that this current injury has not decreased as much as he would have expected over the past two weeks.

Paul has not seen a GP for several years and he is single and lives by himself. He drinks the occasional beer and doesn't smoke.

Pauls' examination results are outlined in the table below:

Examination:

Vitals	Height 178cm<br>Weight 88kg<br>BMI 27.7<br>Blood pressure 135/85 Heart rate 76
Postural analysis	Decreased lumbar and thoracic curves with forward head carriage.<br>Left elevated pelvis with superior adductor notch, and flattening of the superolateral gluteal region on the same side. Bilateral bulking of the hamstrings.<br>Bilateral pes planus.
Range of Motion	Left hip: decreased active abduction (with pain) and flexion (no pain), mild discomfort on active adduction; decreased passive abduction (with pain) and flexion (with a hard end feel); pain on resisted adduction<br>Right hip: decreased hip flexion actively and passively with a hard end feel<br>Lumbar: decreased active extension and right lateral flexion (no pain)<br>Knee: normal range of motion bilaterally
Muscle assessment	Shortness of the bilateral hamstrings, bilateral lumbar erector spinae, left quadratus lumborum and right tensor fascia lata. Left adductor length assessment limited by pain with both straight knee and bent knee;<br>Tenderness to palpation, and hypertonicity, of the left hip adductors (tenderness close to the pelvis insertion point), left gluteus medius, left quadratus lumborum, and right piriformis;<br>Pain on strength test left hip adductors in medial hip with +4 strength, compared to no pain and +5 strength on right

Joint assessment	Tenderness on prone springing of L4 vertebrae<br>Joint restrictions: L4 right rotation and left lateral flexion restriction; Right sacroiliac joint extension restriction; Right pubis superior to inferior glide restriction; Bilateral hip flexion (with compression) restriction (no pain) and left hip long axis restriction (some pain on springing); Left knee lateral glide restriction
Orthopaedic/Neurologic assessment	Lumbar compression/distraction: negative<br>SLR: limited to 70 degrees bilaterally but no pain or symptoms<br>Sacroiliac compression: restricted on right but no pain or tenderness<br>Gaenslens: negative<br>Fabere: limited range with pain in the medial hip on the left Hip scour test: bilaterally restricted in flexion but no pain on compression<br>Trendelenburg positive on left with pain in medial hip
Functional assessment	Active straight leg raise: pelvic torsion noted bilaterally Hip abduction: early hike of pelvis on left with pain in the medial hip at full range; right normal<br>One leg standing: 10 seconds on right, no pelvic shift; couldn't assess objective component on left due to hip pain, right pelvis dropped<br>Squat: discomfort in left medial hip during movement, worse straightening up; lumbar flexion, and increased left knee valgosity observed

Paul was given the following diagnosis:

Acute grade 1 muscle strain of the left hip adductor muscles with associated sacroiliac joint dysfunction. Predisposed by weak left gluteus medius with compensatory short left quadratus lumborum muscle. Complicated by increased stress at work, decreased exercise and possible hypertension.

Question 1:

Based on the above information, please outline a detailed 6-week management plan for this patient.

Case Study 2

Michelle, a 66-year-old retired lawyer presents with an eight week history of increasing left ankle pain. Michelle is uncertain how the pain started but feels quite stiff through the back of her ankle when getting up after sitting or sleeping, and she finds it quite uncomfortable to walk up and down stairs. The pain hasn't really changed over the last eight weeks and she rates it as a four on a ten point scale at its worst.

She describes the pain as an achy or stiff feeling that is there most of the time. It gets worse when she walks, particularly when she'd been sitting down for a while or when she first gets up in the morning. The pain in the left heel gets a bit sharper at that time and she feels like she's hobbling for the first few steps as she gets going. As she continues to walk it feels a bit easier, but if she is on her feet a lot it feels worse by the end of the day. The pain is aggravated the most when she has to walk up or down stairs or hills. She also finds that it feels worse in the morning when she has slept on her stomach at night. Rest helps to relieve the pain and she also finds walking easier if she wears shoes with a slight heel. She hasn't tried any heat, ice or pain-killers. The pain is localised to the back of her left ankle, just above the heel bone and she doesn't report any associated numbness, pins and needles or weakness. Michelle reports that she has also been concerned about swelling of both her ankles that she has noticed over the past few months. The swelling increases towards the end of the day and she finds it's worse if she doesn't wear supportive shoes.

On systems review Michelle reports that she has been struggling with some general fatigue for several months but she puts it down to poor sleep as she finds she needs to urinate several times a night, and then finds it difficult to get back to sleep. She says her ankle pain has not made sleeping more difficult. She used to get out of the house for half hour walks two to three times a week but recently she hasn't been walking as much as she doesn't want to aggravate her ankle. She has been trying to decrease her weight recently but she finds it hard to stick to her dietary changes and now she can't exercise as much either. She has not been diagnosed with any chronic conditions and she last visited the GP several months ago. Michelle lives with her husband, she has two adult children who are both currently overseas.

Michelles' examination results are outlined in the table below:

Examination:

Vitals	Height 168cm<br>Weight 84kg<br>BMI 29<br>Blood pressure 130/80 Heart rate 72
Postural analysis	Increased lumbar and thoracic curves with forward head carriage<br>Anterior drawn posture from the ankles (ankle dorsiflexion)<br>Flattening of the superolateral gluteal region bilaterally<br>Prominence of the thoracolumbar erector spinae bilaterally<br>Slight flexion of the left knee<br>Bilateral pes planus, worse on left
Range of Motion	Left ankle: Decreased active and passive dorsiflexion with pain; mild pain on resisted pantarflexion<br>Right ankle: Decreased active and passive dorsiflexion with no pain<br>Knees: Normal range bilaterally<br>Hips: Normal range bilaterally<br>Lumbar: Decreased flexion with no pain
Muscle assessment	Shortness and hypertonicity of the bilateral hamstrings, bilateral psoas, bilateral lumbar erector spinae, and bilateral gastrocnemius with pain at end range and on springing on the left;<br>Tenderness to palpation, over the left Achilles tendon just proximal to its insertion into the posterior calcaneus<br>Pain on strength test left gastrocnemius with +4 strength, compared to no pain and +5 strength on right

Soft tissue observation and	Generalised swelling around both ankles, non-tender except over the Achilles tendon on the left. No tenderness either side of the Achilles tendon bilaterally. No colour change or trophic change in the feet or

palpation	toes.
Joint assessment	Tenderness on prone springing of T12 and L4<br>Joint restrictions: T12 extension restriction, L4 right rotation and left lateral flexion restriction; Left knee lateral glide restriction; Left calcaneal eversion restriction, Left superior tibiofibular joint P-A glide restriction, Left talotibial A-P glide restriction
Orthopaedic/Neurologic assessment	Lumbar compression/distraction: Negative<br>SLR: Limited to 70 degrees bilaterally but no pain or symptoms<br>Sacroiliac compression: Negative<br>Gaenslens: Negative<br>Fabere: Negative<br>Ankle eversion and inversion stress tests: Negative Homan's sign: Positive on the left for pain Thompson's squeeze test: Negative
Functional assessment	One leg standing: 10 seconds bilaterally with lateral pelvic shift on both sides, some discomfort when performing on left<br>Squat: Discomfort in left ankle during movement; limited squat range observed before heels started to lift from floor<br>Hip extension movement pattern: Late gluteal activation bilaterally with increased lumbar lordosis<br>Hip flexion movement pattern: Heels lifting off the table as flexion initiated<br>Active straight leg raise: Pelvic torsion bilaterally

Michelle was given the following diagnosis:

Subacute left Achilles tendinosis with associated shortness of the gastrocnemius and restricted ankle and hindfoot joint movement. Predisposed by anterior drawn posture, poor lumbopelvic stability, and reduced proprioception. Complicated by increased weight and sedentary lifestyle, and possible onset of secondary diabetes mellitus.

Question 1:

Based on the above information, please outline a detailed 6-week management plan for this patient.

Case Study 3

Catherine, a 24-year-old part-time university student presents with a six week history of increasing right knee pain. Catherine plays volleyball competitively at the state level and trains three times a week (two hour sessions) and plays twice a week. She reports that about six weeks ago she started getting some knee pain after training and games, however, now the pain is more constant and she is worried it is going to start impacting on her ability to continue playing volleyball.

Originally the pain would come on shortly after a game or a heavy training session and last for a few hours. She would find that she had no difficulty sleeping and there would be no pain when she woke the next day. She continued playing and training as usual but in the last 2 weeks the pain has become more noticeable. She now feels pain in her right knee when she is playing, and particularly tends to get a sharp twinge when she jumps. Her knee is then very achy after playing and she can still feel some discomfort the following day. It still doesn't affect her sleep. She rates the ache as a two on a ten point scale, going up to a six out of ten with the sharp twinges. She reports she is still able to play as she normally would, but she is less keen to do jumps and squats in training due to the sharper aggravations of pain. The pain is localised to just under her kneecap and she reports that the location hasn't changed. There is no associated numbness, pins and needles or weakness. She has been using ice over the area after training sessions and games which she feels helps a bit, and she has been taking nurofen before games over the last couple of weeks to try and prevent the pain from coming on. She is uncertain if that has helped – she still felt some pain during the games but it may have been less than if she hadn't used the nurofen.

On systems review Catherine reports that she occasionally feels anxious and can feel her 'heart beating loudly' but the feeling doesn't persist. She has irregular periods, often going many months without menstruating, however, she isn't particularly concerned about this and hasn't had it assessed. She says her diet is good as she is very careful to keep her calorie intake low to maintain her current weight. She drinks and smokes socially on weekends or at parties. She lives at home and is studying part-time so she has time for her sporting commitments.

Catherines' examination results are outlined in the table below:

Examination:

Vitals	Height 182cm<br>Weight 57kg<br>BMI 17<br>Blood pressure 115/80 Heart rate 68
Postural analysis	Decreased lumbar and thoracic curves with forward head carriage<br>Bilateral bulking of the hamstrings<br>Flattening of the superolateral gluteal region bilaterally Low PSIS and high iliac crest on the right<br>Bilateral genu valgus<br>Bilateral pes planus
Range of Motion	Right knee: Discomfort on full active and passive flexion, mild pain on resisted extension;<br>Left knee: Full active and passive range with no pain; Hip: Normal range bilaterally;<br>Lumbar: Decreased extension with no pain;<br>Ankle: Decreased passive ankle dorsiflexion bilaterally
Muscle assessment	Shortness and hypertonicity of the bilateral hamstrings, bilateral gastrocnemius, bilateral rectus femoris (right with some discomfort on springing), and right tensor fascia lata; Tenderness to palpation, over the right patella tendon close to patella insertion with mild swelling noted in the area around the proximal tendon;<br>Pain on strength test right rectus femoris with +4 strength, compared to no pain and +5 strength on left
Joint assessment	Tenderness on prone springing of right sacroiliac joint<br>Joint restrictions: Generalised extension restrictions lumbar spine; Right sacroiliac joint extension restriction; Right knee lateral glide restriction; Bilateral navicular plantar to dorsal restriction

Orthopaedic/Neurologic assessment	Lumbar compression/distraction: Negative<br>SLR: Limited to 70 degrees bilaterally but no pain or symptoms<br>Sacroiliac compression: Tender on right<br>Gaenslens: Negative<br>Fabere: Negative<br>Hip scour test: Negative<br>McMurray's test: Negative<br>Lachman test: Negative<br>Knee effusion test: Negative<br>Noble compression test: Negative<br>Posterior sag sign: Negative
Functional assessment	One leg standing: 10 seconds bilaterally with lateral pelvic shift on both sides<br>Squat: Discomfort in right knee during movement, worse straightening up; lumbar flexion, knees in front of toes, increased pes planus, and increased bilateral knee valgosity observed<br>Hip extension movement pattern: Late gluteal activation bilaterally with flexed knee on the right<br>Hip flexion movement pattern: Normal<br>Lunge: Increased knee valgosity, pes planus, and knees in front of toes bilaterally but more pronounced on the right. Increased right anterior knee pain towards full lunge on right

Catherine was given the following diagnosis:

Subacute right patellar tendinosis secondary to repetitive strain from volleyball. Predisposed by faulty lower limb biomechanics including pes planus, genu valgus and reduced proprioception, with associated right hamstring overactivity and right sacroiliac joint restriction. Complicated by low body mass and anxiety.

Question 1:

Based on the above information, please outline a detailed 6-week management plan for this patient.
