# Supplementary material for: The usefulness of a novel patient management decision aid to improve clinical decision-making skills in final year chiropractic students
Source: Chiropr Man Therap. 2019 Sep 19;27:55. doi: 10.1186/s12998-019-0278-3 (PMC6751823; doi:10.1186/s12998-019-0278-3)
Supplement: Supplementary file 5 — Post-initial exposure questionnaire. (PDF 72 kb) [file 12998_2019_278_MOESM5_ESM.pdf]

Additional File 5

Post-Initial Exposure Questionnaire

Research Participant Questionnaire

Section 1: Demographic details

Please answer the following questions:

1. What is your age? _________years

2. What is your gender? ___________

Section 2: Usability of algorithm

We would like to ask you some questions about the algorithm that you have recently used. Please check the box that reflects your immediate response to each statement. Don't think too long about each statement. Make sure you respond to every statement. If you don't know how to respond, simply check box "3".

1. I think I would like to use this decision aid frequently	1	2	3	4	5
2. I found the decision aid unnecessarily complex	1	2	3	4	5
3. I thought the decision aid was easy to use	1	2	3	4	5
4. I think that I would need the support of an educator to be able to use this decision aid	1	2	3	4	5
5. I found the various elements in the decision aid were well integrated	1	2	3	4	5
6. I thought there was too much inconsistency in this decision aid	1	2	3	4	5

7. I imagine that most people would learn to use this decision aid very quickly	1	2	3	4	5
8. I found the decision aid very awkward to use	1	2	3	4	5
9. I felt very confident using the decision aid	1	2	3	4	5
10. I needed to learn a lot of things before I could get going with this decision aid	1	2	3	4	5
11. I felt more confident in my responses when I used the decision aid	1	2	3	4	5
12. Using the decision aid did not improve my decision-making ability	1	2	3	4	5
13. Using the decision aid helped me to organise and structure my responses	1	2	3	4	5
14. The decision aid was useful as a memory aid when formulating my responses	1	2	3	4	5

Please provide any further comments that you have regarding the algorithm that you recently used:

____

____

Thank you for completing this questionnaire. We value your time and appreciate that you have contributed to our research.
