# Supplementary material for: The usefulness of a novel patient management decision aid to improve clinical decision-making skills in final year chiropractic students
Source: Chiropr Man Therap. 2019 Sep 19;27:55. doi: 10.1186/s12998-019-0278-3 (PMC6751823; doi:10.1186/s12998-019-0278-3)
Supplement: Supplementary file 6 — Post-repeated exposure questionnaire. (PDF 84 kb) [file 12998_2019_278_MOESM6_ESM.pdf]

Additional File 6

Post-Repeated Exposure Questionnaire

Research Participant Questionnaire

Section 1: Demographic details

Please answer the following questions:

1. What is your age? _________years

2. What is your gender? ___________

Section 2: Usability of algorithm

We would like to ask you some questions about the algorithm that you have recently used. Please check the box that reflects your immediate response to each statement. Don't think too long about each statement. Make sure you respond to every statement. If you don't know how to respond, simply check box "3".

1. I found that the algorithm helped me to remember the different components of a management plan.	1	2	3	4	5
2. Using the algorithm throughout the semester did not improve my ability to formulate a patient management plan.	1	2	3	4	5
3. I found that using the algorithm improved my ability to integrate various management techniques learnt across different units of study.	1	2	3	4	5
4. I found that the algorithm was integrated well within the case-based learning tutorials.	1	2	3	4	5
5. I think that using the algorithm throughout the semester improved my ability to formulate a patient management plan within a clinical setting (i.e. at the student clinic).	1	2	3	4	5
6. I think that using the algorithm throughout the semester improved my ability to formulate a patient management plan within an exam.	1	2	3	4	5
7. I was already very familiar with all the components of a management plan and did not need to use the algorithm to remember all the components.	1	2	3	4	5

8. Using the algorithm throughout the semester hampered or hindered my ability to formulate a patient management plan.	1	2	3	4	5
9. I found that using the algorithm within case-study examples throughout the semester helped me to understand how to use the algorithm.	1	2	3	4	5
10. Using the algorithm throughout the semester did not help me to integrate various management techniques learnt across different units of study.	1	2	3	4	5
11. I think case-based learning with the use of the algorithm should be continued in the second semester of 2017.	1	2	3	4	5

1) What do you consider most helpful about the algorithm?

____

____

____

2) What do you think is missing from the algorithm?

____

____

____

3) How would you improve the algorithm?

____

____

____

4) Please provide any further comments that you have regarding the algorithm that was used during the semester:

____

____

____

____

Thank you for completing this questionnaire. We value your time and appreciate that you have contributed to our research.
