# Supplementary material for: The usefulness of a novel patient management decision aid to improve clinical decision-making skills in final year chiropractic students
Source: Chiropr Man Therap. 2019 Sep 19;27:55. doi: 10.1186/s12998-019-0278-3 (PMC6751823; doi:10.1186/s12998-019-0278-3)
Supplement: Supplementary file 7 — Standardised marking rubric for case-based assessment tasks. (PDF 122 kb) [file 12998_2019_278_MOESM7_ESM.pdf]

Additional File 7

Standardised Marking Rubric for Case-Based Assessment Tasks

Section	0	1	2	3
**Prognosis**	Not addressed	Basic prognosis given, little consideration of case specific factors	Good consideration of case specific factors in prognosis	
**Goals/Schedule**	Not addressed	Basic response of both goals and schedule or only one done in detail	Good consideration of case specific factors in goals and scheduling	
**Advice**	Not addressed	Mentioned but not in clinical context or sufficient detail	Good demonstration of clinically relevant advice but not all components given	Good demonstration of clinically relevant advice of all components (including reassurance)
**Passive**	Not addressed	Very limited clinical detail (ie. soft tissue work and adjust as indicated)	Good delineation of relevant techniques but without sufficient detail to be able to repeat treatment elements	Sufficient clinical detail to be able to repeat treatment
**Active**	Not addressed	Very limited clinical detail (ie. perform core strength exercises)	Good delineation of relevant techniques but without sufficient detail to be able to repeat exercises	Sufficient clinical detail to be able to repeat exercises
**Preventative**	Not addressed	Mentioned but not in clinical context or sufficient detail	Good level of detail and clinically relevant	
**Reassessment**	Not addressed	Mentioned but not in clinical context or sufficient detail	Clinically relevant tag tests and end of care reassessment outlined	
**Clinical Reasoning**	None	Poor - Basic clinical reasoning demonstrated	Basic - Good clinical reasoning demonstrated	Excellent clinical reasoning demonstrated
