# Supplementary material for: The usefulness of a novel patient management decision aid to improve clinical decision-making skills in final year chiropractic students
Source: Chiropr Man Therap. 2019 Sep 19;27:55. doi: 10.1186/s12998-019-0278-3 (PMC6751823; doi:10.1186/s12998-019-0278-3)

Additional File 8

Interpreting the System Usability Scale (SUS) score according to Bangor et al., 2008 (reproduced with permission).

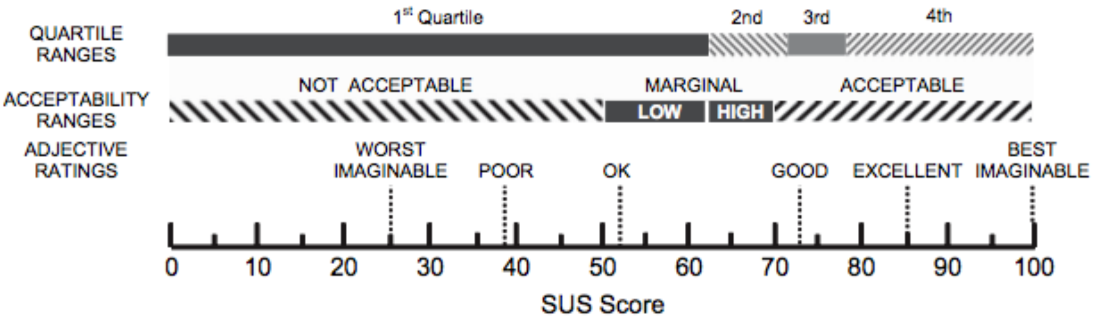

Supplement: Supplementary file 8 — Interpreting the System Usability Scale (SUS) score according to Bangor et al., 2008 (reproduced with permission) [19]. (PDF 77 kb) [file 12998_2019_278_MOESM8_ESM.pdf]
